# A novel molecular synchrotron for cold collision and EDM experiments

**DOI:** 10.1038/srep32663

**Published:** 2016-09-07

**Authors:** Shunyong Hou, Bin Wei, Lianzhong Deng, Jianping Yin

**Affiliations:** 1 State Key Laboratory of Precision Spectroscopy, East China Normal University, Shanghai 200062, P. R. China

## Abstract

Limited by the construction demands, the state-of-the-art molecular synchrotrons consist of only 40 segments that hardly make a good circle. Imperfections in the circular structure will lead to the appearance of unstable velocity regions (i.e. stopbands), where molecules of certain forward velocity will be lost from the structure. In this paper, we propose a stopband-free molecular synchrotron. It contains 1570 ring electrodes, which nearly make a perfect circle, capable of confining both light and heavy polar molecules in the low-field-seeking states. Molecular packets can be conveniently manipulated with this synchrotron by various means, like acceleration, deceleration or even trapping. Trajectory calculations are carried out using a pulsed ^88^SrF molecular beam with a forward velocity of 50 m/s. The results show that the molecular beam can make more than 500 round trips inside the synchrotron with a 1/e lifetime of 6.2 s. The synchrotron can find potential applications in low-energy collision and reaction experiments or in the field of precision measurements, such as the searches for electric dipole moment of elementary particles.

Spectacular success has been made recently on manipulation of polar or paramagnetic molecules by using time-varying electric or magnetic fields^1^,^2^,^3^. A variety of techniques, such as Stark/Zeeman deceleration^4^,^5^,^6^,^7^,^8^, electric/magnetic trapping^9^,^10^,^11^,^12^ and storage ring/synchrotron^13^,^14^,^15^, have well been established to manipulate molecules in both position and velocity space, paving the way for many precision experiments ranging from high-resolution spectroscopy^16^,^17^, molecular collisions^18^,^19^,^20^,^21^,^22^,^23^ to EDM measurement^24^. Among the above mentioned methods, synchrotrons have many advantages in molecular experiments, like (i) the molecular packet keeps its high density while revolving in the synchrotron due to 3D (three-dimensional) confinement; (ii) the stored packet can make its repeated appearance at well-defined times and positions; (iii) multiple packets can be injected into the synchrotron, which can enhance the number of collisions; (iv) suitable for low-energy collision study since collision partners spend long time together when they are in the same traveling potential well.

In 2001, the first prototype storage ring for neutral molecules, proposed by D. P. Katz^25^, was demonstrated by Crompvoets *et al*.^13^. This storage ring is made of a hexapole torus and allows for a molecular packet of ammonia traveling up to six round trips. Later, a sectioned storage ring consisting of two semicircle hexapoles was investigated^14^. The confined molecules made 100 round trips in the ring, corresponding to a flight distance of 80 m. More recently, a synchrotron consisting of 40 straight hexapoles has greatly improved the number of stored molecular packets up to 19 and their revolving distance over 1 mile^15^. Some other molecular storage ring/synchrotron designs were also proposed, either for low-field seekers^26^,^27^ or for high-field seekers^28^,^29^. Most recently, a scheme for a magnetic molecular synchrotron composed by 40 hybrid magnetic hexapole lenses was presented as well^30^.

Molecules in a storage ring might have their oscillatory amplitude enlarged due to imperfections in the circular structure and get lost. This phenomenon is termed as motional resonances^31^. It occurs only for certain combinations of the molecular velocity and applied voltage. These unstable velocity regions are called “stopbands”^32^. The currently realized molecular synchrotrons, however, consist of only 40 segments or less and thus hardly make a good circle. As a result, appearance of stopbands will happen in these devices. Additionally, inside these synchrotrons the longitudinal bunching force for molecules stems from fringe effects of the used electric/magnetic fields, which is usually insufficient to manipulate heavy particles. In this paper, we propose a 1570-segment synchrotron capable of addressing the above mentioned two problems. Our proposed synchrotron is formed by placing an array of metal flat rings along a torus with a diameter of 1 m. The large number of segments makes it a nearly perfect circle, avoiding unstable velocity regions, i.e. stopbands. Our proposed synchrotron works in a manner similar to the traveling wave Stark decelerator, and yields a series of true three-dimension (3D) potential wells within the structure, capable of confining both light and heavy polar molecules. In the following sections, we will first give a brief introduction to the design and operation principle of the proposed synchrotron. Then a detailed analysis on the longitudinal motion of molecules and their transverse stability will be presented. After that 3D trajectory calculations of SrF confined in the synchrotron will be performed, which is followed by some discussion. Some main results and conclusions will be given in the end.

## Theory

### Design and operation principle

**Figure 1:**
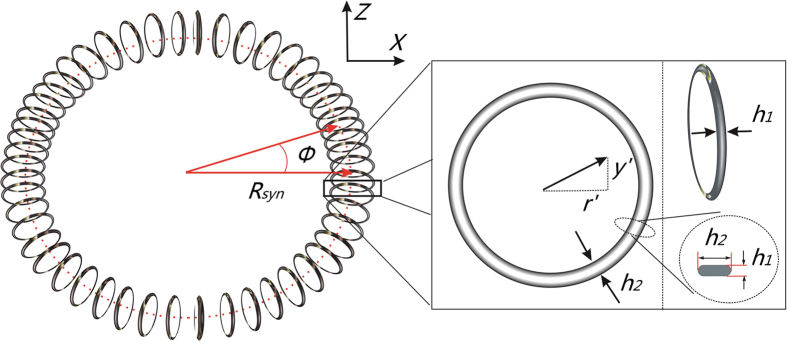
Schematic of the molecular synchrotron, together with the coordinates used in this paper. The position of a molecule is indicated with three coordinates *ϕ* (tangential coordinate), *r*′ (radial coordinate) and *y*′ (vertical coordinate). Not all rings are illustrated. The inset on the most right shows the cross section of an individual flat ring, where the thickness *h*_*1*_ = 0.6 mm and the width *h*_*2*_ = 3.6 mm.

The scheme of the synchrotron is shown in Fig. 1. It is composed by 1570 metal flat rings arranged in a circle with a radius of 0.5 m. Each ring is formed from a flat wire of thickness *h*_*1*_ = 0.6 mm and width *h*_*2*_ = 3.6 mm, and has an inner diameter of 10 mm. The distance between two neighboring rings is *L* = 2.0 mm with a gap of 1.4 mm between them. The synchrotron works in a way similar to the traveling wave Stark decelerator^33^,^34^,^35^,^36^,^37^,^38^. All rings in the synchrotron are periodically connected in 8 sets to 8 high-voltage supplies, which are spatially sine-modulated and expressed as 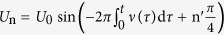, where n′ = 1, …, 8 is the number of the electrode, and *U*_0_ and *ν*(τ) are the waveform amplitude and modulation frequency of the voltage on each individual electrode, respectively. In this way, an array of periodic 3D potential wells for polar molecules of low-filed-seeking states is formed inside the synchrotron. The potential wells move smoothly with a speed of *Lν*(τ) along the tangential direction. The velocity of the potential well can be changed by chirping the modulation frequency. Since it is difficult to obtain an analytical expression of the electric fields in the synchrotron, we turn to the numerical calculations. The transverse dimension *r*_*0*_ of the rings is far less than the curvature radius *R*_*syn*_ of the synchrotron, whose electric field is only so slightly distorted from a straight one that it can be taken as a traveling wave decelerator arranged in a circle. Both the electric field configuration and the operation principle of our synchrotron are similar to the traveling wave decelerator^33^,^38^.

### Stark shift of SrF

**Figure 2:**
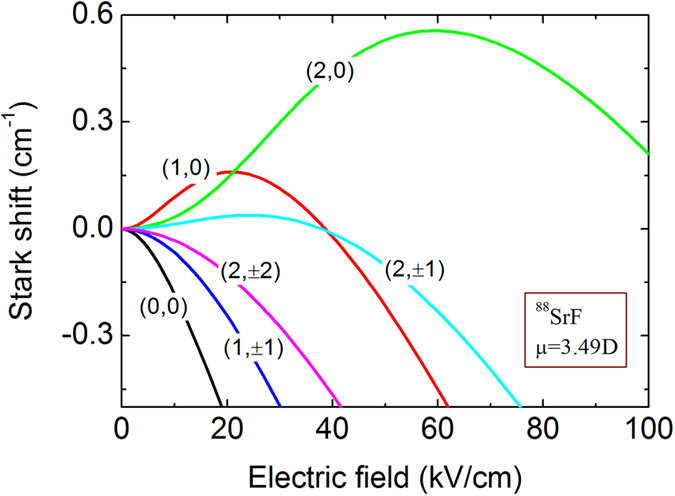
Stark shift of ^88^SrF of low-lying rotational states (*N, N*_*M*_), where *N* is the rotational number and *N*_*M*_ is the projection of *N* on the electric field axis.

^88^SrF molecule is chosen as a tester to validate our proposed synchrotron, because it is a typical heavy polar molecule (roughly defined as mass >100 amu) suited for measurement of parity violation^39^,^40^,^41^,^42^,^43^, and because it is amenable to laser cooling^44^. Figure 2 shows the Stark shift of SrF of lowest rotational levels (X^1^Σ^+^, *N* = 0, 1, 2) in the vibronic ground state, where *N* is the rotational quantum number. The (*N, N*_*M*_) = (2, 0) state is selected for the following studies, where *N*_*M*_ is the projection of *N* on the electric field axis. The force experienced by a SrF molecule is derived from the Stark potential utilizing the formula


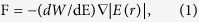


where *W* is the Stark potential energy and E is the electric field strength.

### Longitudinal confinement

**Figure 3:**
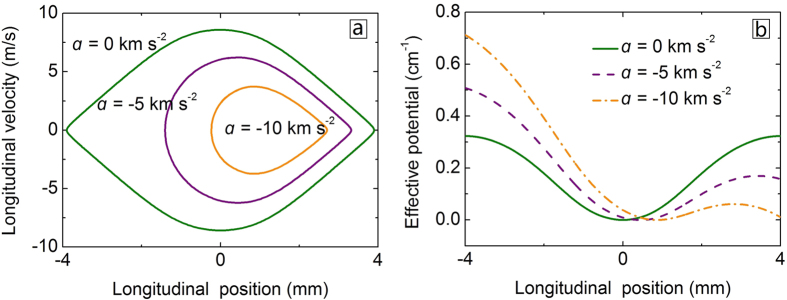
Phase space seperatrices (**a**) and effective potential wells (**b**) for SrF of the (*N, N*_*M*_) = (2, 0) state in the synchrotron for three selected accelerations.

The motion of a molecule relative to the moving potential well in the synchrotron is given by^45^


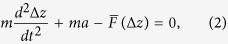


where Δ*z* is the longitudinal position difference between the molecule and the potential well center, *a* is the acceleration of the potential well, and 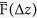 is the average force of the nonsynchronous molecule in the potential well over one period. 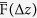 can be expressed as a Fourier series


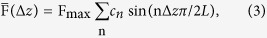


where *L* is the distance between adjacent electrodes, and the *c*_n_ are the coefficients depending on the Stark shift of the molecule and the synchrotron. For a good approximation, we retain the first fifteen terms in our calculations for SrF in the (*N, N*_*M*_) = (2, 0) state. By integrating the equation (2), the phase stability diagrams of the synchrotron can be obtained. Using the parameters listed in Table 1, the separatrices and effective potentials for SrF molecule with different accelerations are shown in Fig. 3. It is clear that with increasing acceleration, the seperatrix gets smaller and the depth of the potential becomes shallower, implying less molecules to be captured by the synchrotron. The center of the potential well also shifts forward. The depth of the potential well of our synchrotron is about 0.5 K when *a* = 0 km s^−2^, which is more than one order of magnitude larger than those of previous versions^14^,^15^. This means larger acceleration and acceptance can be offered by our synchrotron.

### Transverse effective trap

The potential wells formed in the synchrotron are nearly cylindrical along the beam axis and the electric fields increase radially apart from the axis. Molecules in the low-field-seeking states are focused in both transverse directions inside the synchrotron. Because the transverse potential changes in time, we introduce an averaged transverse force to characterize the transverse motion of molecules. It is obtained by integrating the radial force along the structure over one period and expressed as


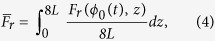


where *z* is the longitudinal position, and *ϕ*_0_(*t*) is the time-dependent phase offset expressed as 
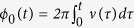
. It is worth nothing that the averaged transverse force is weakly dependent on the acceleration of the synchrotron and offers the centripetal force to keep the molecule in an equilibrium radius. If the averaged force is linear, the equilibrium can be written as,


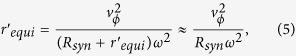


where *ω* is the betatron frequency and 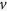
_*ϕ*_ is the tangential velocity of the molecule. Using the averaged transverse force, we get the mean angular oscillation frequency for SrF molecule, ω_r_/2*π = *319 Hz, under the conditions of Table 1. The pseudo-potential energy contributed by the centrifugal force is given by:


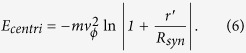


The effective potential energy for a molecule in the synchrotron, *E*_tatal_, is the sum of the pseudo-potential energy and the Stark energy,


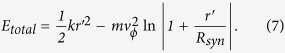


**Figure 4:**
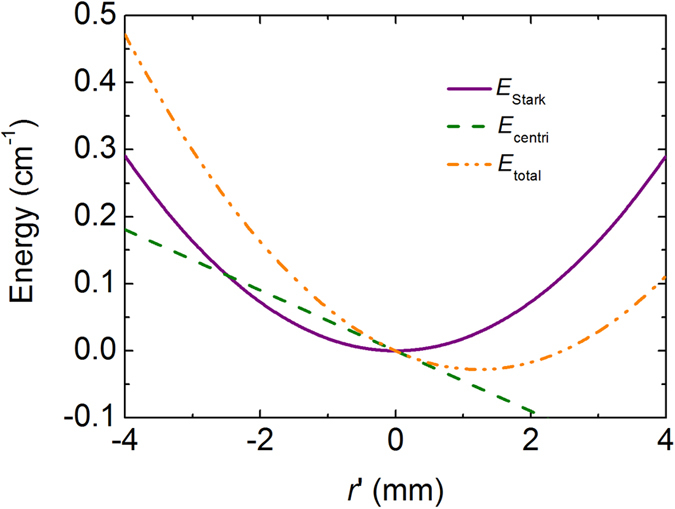
The radial position dependence of the effective energy (dash-dotted curve) for a SrF molecule with a forward velocity of 50 m/s. The pseudo-potential energy (dashed curve) and the Stark energy (solid curve) are also shown for comparison.

The dash-dotted curve in Fig. 4 shows the effective potential energy for a SrF molecule as a function of radial position under the conditions of Table 1. The forward velocity of molecule is set to be 50 m/s. For comparison, the pseudo-potential energy (dashed curve) and the Stark energy (solid curve) are also presented in the figure. With increasing forward velocity the effective potential well becomes shallower and the equilibrium orbit shifts outwards as well.

### Transverse stability

As mentioned before, low-degree symmetry of a synchrotron will lead to the appearance of stopbands, where molecules with certain forward velocity will loss from the structure^31^. One method to avoid stopbands is increasing the number of segments, i.e., improving the symmetry of the synchrotron. Our proposed scheme of synchrotron contains as many as 1570 segments, forming a good circle. The idea of our scheme is inspired by the traveling wave Stark decelerator^33^, which permits to be constructed with a huge number of electrodes^37^. A traveling wave decelerator composed of 1300 electric rings has been experimentally demonstrated. What’s more, a much longer one including as many as 3200 electrodes is under construction^37^. Therefore our synchrotron consisting of 1570 elements is practicable in manufacture.

**Figure 5:**
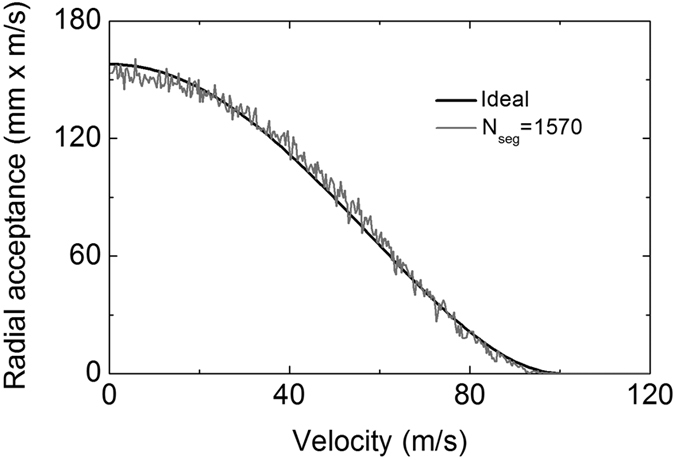
The radial acceptance for the synchrotron consisting of 1570 segments (gray) as a function of the forward velocity, together with the acceptance for an ideal ring (black).

In order to confirm the transverse stability of the synchrotron, we take a trajectory simulation following the method given by Heiner^46^ and Zieger *et al*.^47^. The initial position and velocity distributions of the packet are Gaussian with FWHM (full width at half maximum) of [10 mm × 20 m/s]. This selected molecular phase space is larger than the transverse acceptance of the synchrotron. The parameters of the synchrotron are listed in Table 1. Since the transverse force on the molecules in the synchrotron changes continuously in time, the averaged force over one period is taken as an input. We start the simulation with 2000 molecules and check whether the molecules have escaped from the synchrotron at every step. The acceptance is obtained by comparing the fraction of molecules survived in the synchrotron after 200 ms to the number of molecules initially captured by the synchrotron. The calculated results are shown in Fig. 5. The gray line indicates the phase-space acceptance for the 1570-segment synchrotron as a function of the forward velocity, while the black curve illustrates the acceptance for an ideal ring. The radial acceptance for the synchrotron is actually identical to the ideal case, where the acceptance for the ideal ring is calculated from the following expression^47^:


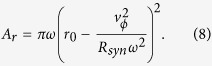


This formula is hold when the restoring force is linear. As can be seen from Fig. 5, no stopbands appear in the 1570-segment synchrotron, which explicitly shows the transverse stability of molecules within the structure. Without stopbands, the forward velocity of the packets can be continuously changed, which is essential to measure the resonances in inelastic scattering experiments. Note that in our simulation nonadiabatic loss around the center of the synchrotron is not considered. Therefore the calculated acceptance is overestimated at low velocities.

## Results

### The lifetime of SrF molecules in the synchrotron

A storage ring (or a trap) offers particles an area thermally isolated from the outside world, and the length of storage time is crucial for experiments involving long-lived effects in molecular system. Longer confinement times are pursued by various experimental studies. For instance, (i) direct lifetime measurements of vibrational levels of molecules usually require 1 ms−1 s, which is hardly realized in beam experiments, but easily in a trap^48^,^49^; (ii) the most recently developed laser cooling technique for polyatomic molecules will take over 10 seconds to cool down the temperature of the molecules^50^,^51^; (iii) the state-selected molecular collision experiments thus far have been limited by low number density of colliding partners. The density limitation, however, can be overcome by storing many molecular packets in a synchrotron over an increased time^47^,^52^; (iv) the statistical sensitivity of precision measurement of fundamental constants, like eEDM (electric dipole moment of the electron), might be greatly improved due to the long coherence time of trapped or confined molecules^24^.

**Figure 6:**
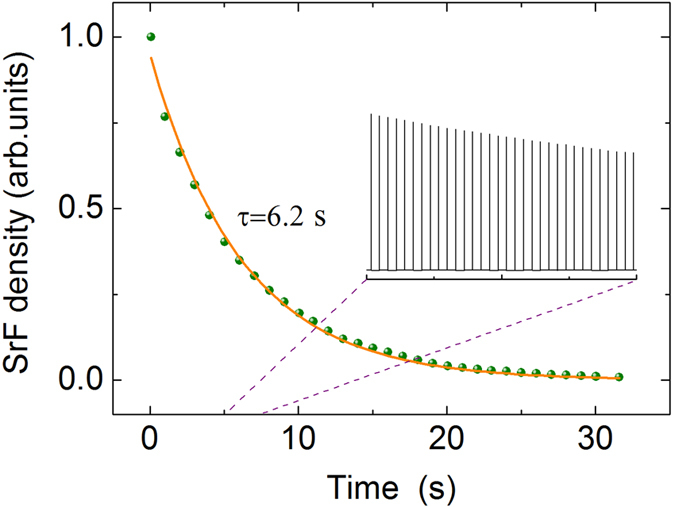
Calculated results of the density of SrF molecules as a function of time (in seconds). It takes some 30 s for the SrF molecular packet with a forword velocity of 50 m/s to travel 500 round trips. The inset typically shows a zoom-in of a part of round trips.

^88^SrF molecule is used to test the validity and the stability of our proposed synchrotron in manipulation of heavy polar molecules. The parameters for the synchrotron are listed in Table 1. SrF in the state (*N* = 2, *N*_*M*_ = 0) is chosen for simulations. Voltage amplitude applied to the synchrotron is 20 kV. The maximum electric field along the beam axis is 32 kV/cm, in which SrF molecules of state (2, 0) remain weak-field seekers. The six dimensional emittance of the input beam is set to be [*Δz* × *Δv*_*z*_] × [*Δx* × *Δv*_*x*_] × [*Δy* × *Δv*_*y*_] = [5 mm × 20 m/s] × [5 mm × 20 m/s] × [5 mm × 20 m/s], where the position and velocity spread are the FWHM of Gaussian distributions. The non-adiabatic loss of the stored molecules is negligible, as demonstrated by Meek and co-workers using a CO molecule beam^38^. The collisional background loss is also neglected in the simulation. Figure 6 shows the calculated results for a SrF molecular pulse revolving in the synchrotron. The molecular packet containing one million molecules has a mean forward velocity of 50 m/s. It takes the SrF molecular packet about 30 s to revolve 500 round trips. As can be seen from this figure, the molecular density decreases monotonously as a function of the revolving time inside the synchrotron. The solid curve is the fitting result, which shows the 1/*e* lifetime of the molecular packet is 6.2 s, which confirms the stability of the synchrotron. The long confinement time shows the validity of our scheme in manipulation of heavy molecules and allows for many exciting molecular studies that involves long interrogation times. Table 2 lists the typical frequencies and acceptance for SrF molecules inside the synchrotron.

### Various manipulations on the synchrotron

**Figure 7:**
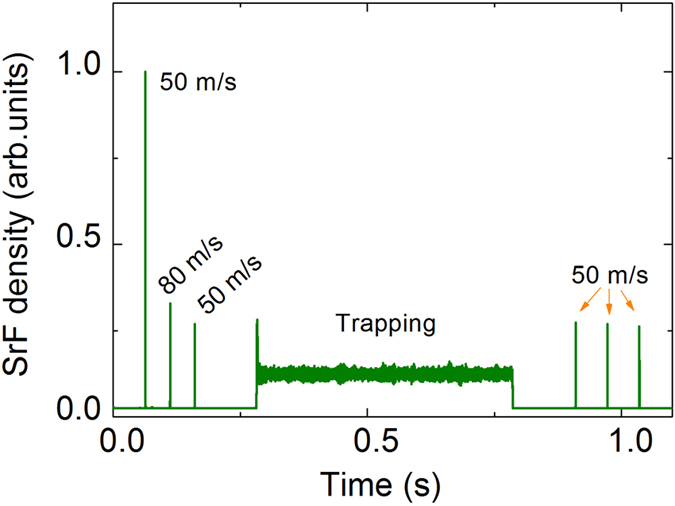
Simulated results of the density of SrF molecular packet revolving in the synchrotron as a function of time under various manipulations, including acceleration, deceleration and trapping.

The strong longitudinal force and transverse stability offered by the synchrotron make it easy to tame polar molecules, even heavy species. In order to show this, we carry out trajectory calculations for SrF molecule under various manipulations, including acceleration, deceleration and trapping. The input molecular parameters are the same as above, except the number of molecule being set to 100 thousand. The simulated results are shown in Fig. 7. The packet of SrF molecles travels at a constant velocity of 50 m/s in the first round trip and then is accelerated to 80 m/s in the second round trip. The molecule number is decreased by 65% in the acceleration process, as can be seen from this figure. The loss is mainly due to the effective transverse potential trap getting smaller and shallower with increasing molecular velocity. In the following two round trips, the packet is lowered from 80 m/s to 50 m/s and then to a standstill inside the synchrotron. Immediately after that, the packet is trapped for 500 ms in the synchrotron. The trapped packet is finally accelerated to 50 m/s again with one more round trip and travels for two additional round trips inside the synchrotron at constant forward velocity of 50 m/s. The density of the packet nearly keeps constant during the trapping time, as can be seen in the figure. This is indicative of the good stability and confinement of the synchrotron.

## Discussion

### Loading

**Figure 8:**
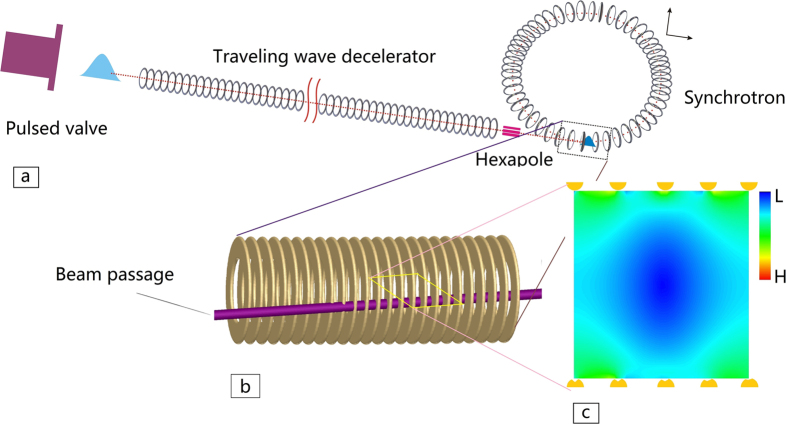
(**a**) schematic view of loading molecular beams into the synchrotron. A beam prepared by a traveling wave decelerator is hexapole coupled into the synchrotron. (**b**) Zoom-in of the section of loading a beam into the device. The beams are coupled into the synchrotron via the beam passage along the tangent of the synchrotron. (**c**) The electric field distribution in the center plane through five damaged electrodes of the synchrotron. Color bar labels H and L mean high and low electric field, respectively.

A typical loading scheme is shown in Fig. 8. A supersonic or subsonic molecular beam is firstly slowed down to the desired final velocity by a Stark decelerator, as shown in Fig. 8(a). Then the slowed molecular packet is hexapole coupled into the synchrotron via a beam passage, formed by a series of holes of diameter 1.6 mm drilled through 15 flat rings, as shown by Fig. 8(b). The reasons for choosing flat rings instead of common rings to form the synchrotron lie in: first, our calculations show that the flat ones can slightly enhance the depth of the potential wells in the transverse direction; second, it is more convenient to drill apertures through the flat rings for loading molecules.

When a molecular packet is travelling in the loading passage, the flat rings with holes are grounded. High voltages are applied to them when the packet reaches the center of the synchrotron. It is worth noting that there are five flat rings damaged in the inner edge by drilling holes. Fortunately, the distribution of the electric fields in the center plane through the five defective electrodes is only slightly affected, as shown in Fig. 8(c). Molecular loss caused by the defective electrodes is very tiny, as confirmed by trajectory calculations. The electric field distribution inside the synchrotron is not affected by the other ten rings damaged in the outer edge or in the middle.

### Applications

Recently developed techniques such as Stark/Zeeman deceleration promise a wealth of opportunities for state-to-state molecular collision experiments, capable of revealing the precise nature of inter- and intramolecular forces at unprecedented level. These methods, however, suffer from disadvantages, like short interaction time or low number density of molecules. Electric/magnetic traps that can offer considerably long interaction time have been exploited in collision studies^20^,^53^. Storage rings/synchrotrons serve as an alternative platform for investigation of cold/ultracold collisions and reactions, benefiting from the advantages mentioned at the beginning of this paper. In our 1570-segment synchrotron, each potential well covers 4 flat rings in space, thus there are, in principle, nearly 400 molecular packets allowed to be confined simultaneously within the structure. Assuming a packet of 3 × 10^7^ molecules emitted from a Stark decelerator is captured and trapped in a single potential well, the total number of stored molecules is ~10^10^ molecules. Therefore, the collisional frequency between the colliding partners can be greatly enhanced. Two other merits of our proposed synchrotron make it ideal for a low-energy collider: (i) the deep potential wells of the synchrotron make it convenient to control the velocity of stored molecules, including heavy molecules; (ii) The absence of stopbands allows for continuous change of the kinetic energy of the collision partners, which is essential to measuring the resonances in inelastic scattering experiments.

The experiments for measuring the electron’s electric dipole moment advance rapidly these years^54^,^55^. Heavy polar molecules are of particular attraction thanks to their high sensitivity for measurement. Since the statistical sensitivity is proportional to coherence time, the electrically confined molecules are anticipated to offer higher sensitivity of measurement than free molecules^24^. For instance, a typical coherence time in the beam experiments is ~0.001 s, while the coherence time in our synchrotron can reach ~10 s, thus the corresponding eEDM measurement might be improved by about two orders of magnitude, as estimated by the equation (7) in ref. 24.

Our proposed synchrotron can also find potential applications in production of cold molecules, such as sympathetic cooling^56^,^57^,^58^,^59^, optoelectrical cooling^50^,^51^ and stochastic cooling of molecules^60^, and so on. If the potential wells inside the synchrotron were fully filled, one can obtain several hundred cold molecular packets at the same time.

## Conclusions

We have proposed a 1570-segment molecular synchrotron with its operation principle similar to the traveling wave decelerator. Its high-degree of symmetry offers stable transverse confinement of molecules without stopbands appearing in current synchrotrons. This merit makes it suitable for a low-energy collider with a continuous change of collision energy. Additionally, the true 3D potential wells and strong longitudinal bunching force in our synchrotron allow for conveniently taming polar molecules, especially heavy ones, which play key roles in many precision measurements. The dynamics of molecules inside the synchrotron were analyzed and trajectory simulations of ^88^SrF molecule were performed. Our studies showed that a packet of SrF molecules with tangential velocity of 50 m/s can have a lifetime of ~6 s in the synchrotron, whose radial and longitudinal acceptances are 6.5 mm × 12 m/s and 8 mm × 17 m/s, respectively.

Our analysis and study with SrF, a typical heavy molecule, are applicable in principle to light polar molecules, who should be more amenable to manipulation in our synchrotron. With all its advantages, our proposed synchrotron can promise a variety of applications ranging from cold collisions to precision measurements, or even in the cooling of molecules. We also noticed that an idea of construction of a molecular synchrotron similar to our version was mentioned by A. Osterwalder *et al*. in ref. 33.

## Additional Information

**How to cite this article**: Hou, S. *et al*. A novel molecular synchrotron for cold collision and EDM experiments. *Sci. Rep.* **6**, 32663; doi: 10.1038/srep32663 (2016).

## Figures and Tables

**Table 1:** List of parameters for the synchrotron.

Parameters	Symbol	value
Radius of the synchrotron	*R*_*syn*_	500 mm
Radius of a segment(inner)	*r*_*0*_	5.0 mm
segment length	*h*_*1*_	0.6 mm
Distance between adjacent segments	*L*	2.0 mm
Voltage amplitude	*U*_*0*_	20 kV
Number of segment	*N*_*seg*_	1570

**Table 2:** Characteristic frequencies and acceptance for SrF molecule in the 1570-segment molecular synchrotron.

Type of motion	Frequency	Acceptance	Trap depth
Cyclotron	*Ω*_*cycl*_/2*π = *16 Hz		
Synchrotron	ω_z_/2*π = *336 Hz	8 mm × 17 m/s	470 mK
Horizontal betatron	ω_r_/2*π = *319 Hz	6.5 mm × 12 m/s	230 mK
Vertical betatron	ω_y_/2*π = *319 Hz	9 mm × 16 m/s	420 mK

The molecule in the (*N, N*_*M*_) = (2, 0) state has a longitudinal velocity of 50 m/s. Voltage amplitude applied to the synchrotron is 20 kV.
